# Mild Behavioral Impairment in Parkinson’s Disease: An Updated Review on the Clinical, Genetic, Neuroanatomical, and Pathophysiological Aspects

**DOI:** 10.3390/medicina60010115

**Published:** 2024-01-07

**Authors:** Efthalia Angelopoulou, Anastasia Bougea, Alexandros Hatzimanolis, Leonidas Stefanis, Nikolaos Scarmeas, Sokratis Papageorgiou

**Affiliations:** 1 Department of Neurology, Aiginition Hospital, National and Kapodistrian University of Athens, 11528 Athens, Greece; angelthal@med.uoa.gr (E.A.); lstefanis@bioacademy.gr (L.S.); ns257@cumc.columbia.edu (N.S.); sokpapa@med.uoa.gr (S.P.); 2 Department of Psychiatry, Aiginition Hospital, National and Kapodistrian University of Athens, 11528 Athens, Greece; alhatzi@med.uoa.gr; 3 Department of Neurology, Columbia University, New York, NY 10032, USA

**Keywords:** mild behavioral impairment, Parkinson’s disease, prodromal Parkinson’s disease, neuropsychiatric symptoms, mild cognitive impairment, cognitive decline, dementia

## Abstract

Neuropsychiatric symptoms (NPS), including depression, anxiety, apathy, visual hallucinations, and impulse control disorders, are very common during the course of Parkinson’s disease (PD), occurring even at the prodromal and premotor stages. Mild behavioral impairment (MBI) represents a recently described neurobehavioral syndrome, characterized by the emergence of persistent and impactful NPS in later life, reflecting arisk of dementia. Accumulating evidence suggests that MBI is highly prevalent in non-demented patients with PD, also being associated with an advanced disease stage, more severe motor deficits, as well as global and multiple-domain cognitive impairment. Neuroimaging studies have revealed that MBI in patients with PD may be related todistinct patterns of brain atrophy, altered neuronal connectivity, and distribution of dopamine transporter (DAT) depletion, shedding more light on its pathophysiological background. Genetic studies in PD patients have also shown that specific single-nucleotide polymorphisms (SNPs) may be associated with MBI, paving the way for future research in this field. In this review, we summarize and critically discuss the emerging evidence on the frequency, associated clinical and genetic factors, as well as neuroanatomical and neurophysiological correlates of MBI in PD, aiming to elucidate the underlying pathophysiology and its potential role as an early “marker” of cognitive decline, particularly in this population. In addition, we aim to identify research gaps, and propose novel relative areas of interest that could aid in our better understanding of the relationship of this newly defined diagnostic entity with PD.

## 1. Introduction

Parkinson’s disease (PD) is the second-most prevalent neurodegenerative disorder after Alzheimer’s disease (AD), affecting approximately 1–1.5% of the elderly population [1]. Neuropathologically, PD is characterized by the dopaminergic neuronal loss in the substantia nigra pars compacta (SNpc), resulting in nigrostriatal degeneration, accompanied by the progressive accumulation of Lewy bodies and Lewy neurites in the remaining neurons [2]. Bradykinesia, resting tremor, rigidity, and postural instability are the primary motor symptoms, while cognitive impairment, neuropsychiatric symptoms (NPSs), sleep disturbances, and autonomic dysfunction constitute some of the most common non-motor manifestations [2]. Mild cognitive impairment (MCI) appears in about one in four patients with PD, representing a prelude to Parkinson’s disease dementia (PDD) with an estimated annual conversion rate of 11% [3]. PDD is one of the core and most burdensome non-motor symptoms of PD, being related to worse quality of life, functional disability, and higher caregiver burden [4,5]. It is predicted that PDD prevalence will triple by 2060 [6], highlighting the need to early detect cognitive decline in PD, identify the related risk factors, and clarify its evolution throughout the course of the disease in terms of clinical, genetic, neuropathological, and pathophysiological aspects.

In this context, the relatively new concept of “pre-PD-MCI” is receiving increasing attention [7]. Several fluid, genetic, and neuroimaging biomarkers have been associated with the development of cognitive decline in PD, such as cerebrospinal fluid (CSF) amyloid β, apolipoprotein E4, single-nucleotide polymorphisms (SNPs) such as BDNF Val/Val and COMT Val/Val, reduced global brain volume, a brain atrophy pattern resembling AD, and reduced uptake of dopamine transporter (DAT) in the caudate nucleus [8,9]. The main risk factors for cognitive impairment in PD include advanced age, male sex, more severe and increased duration of motor impairment, the akinetic-rigid subtype, as well as the presence of REM sleep behavior disorder (RBD) and NPS [10,11].

In PD, cognitive decline and NPSs are closely related at a clinical and pathophysiological level. NPS are core features of the entire course of PD, occurring even in the prodromal and preclinical stages [12]. NPSs become more common as the disease progresses, and approximately nine in ten patients with PD dementia (PDD) experience at least one NPS, with the most frequent of them being depression, anxiety, apathy, and visual hallucinations [13]. In PD, NPSs have been associated with a worse quality of life, increased motor disability, and increased probability of placement in nursing homes [13,14]. Longitudinal studies have shown that individual NPSs, including apathy, visual hallucinations, depression, and anxiety are associated with a higher risk of cognitive decline in PD and conversion from MCI to PDD [15,16,17]. Hence, appropriate behavioral markers represent promising candidates as predictors or early indicators of cognitive decline in PD, which could be used in combination with fluid, genetic, or neuroimaging biomarkers.

The neuroanatomical, pathophysiological, and genetic background of individual NPSs has been extensively investigated in regard to PD. For instance, anxiety and depressive symptoms in PD have been associated with reduced binding of the [(123)I]FP-CIT SPECT tracer to dopamine transporters in the caudate nucleus [18,19]. Depression in PD has been linked to higher medial temporal atrophy, increased progression rate of global cortical atrophy per year [20], and weaker functional connectivity between the left superior temporal gyrus and the right cerebellum posterior lobe [21]. It has been indicated that PD patients with apathy display a widespread decrease in the extra-striatal uptake of dopamine transporters compared to those without apathy, as well as a lower longitudinal dopamine transporter uptake [22]. Abnormal functional connectivity between the anterior cingulate cortex and nucleus accumbens has been demonstrated to precede apathy in patients with PD [23], and apathy has been shown to be 2.6 more common in non-PD individuals carrying GBA mutations compared to healthy controls [24]. Visual hallucinations in patients with PD have also been associated with specific dopamine receptor single-nucleotide polymorphisms (SNPs) [25]. Impulse control disorders (ICDs) in PD have been related to abnormal connectivity between the left anterior putamen andthe left anterior cingulate gyrus and left inferior temporal gyrus [26], while various SNPs may also increase the risk of ICDs in PD, such as serotonin 2A receptor gene (HTR2A) variants [27].

Accumulating evidence suggests that the development of NPSs in the general population of older adults may represent ariskof dementia, even in the absence of cognitive impairment. For instance, depressive symptoms were associated with a higher risk of AD dementia in both older males and females during a 17-year follow-up [28], and apathy in older adults is related to an increased risk of incident probable dementia [29]. Mild behavioral impairment (MBI) is a recently defined neurobehavioral syndrome, characterized by the emergence of persistent and impactful NPSs in non-demented individuals in later life [30]. Similarly to MCI that represents the pre-dementia neurocognitive axis, MBI can be considered as the complementary neurobehavioral risk axis. It reflects a global indicator of behavioral symptoms, and predictor of cognitive decline in the older non-demented population. The presence of MBI has been associated with a higher progression rate to dementia among individuals with MCI or normal cognition [31,32,33,34]. Therefore, MBI is considered as an early clinical marker of dementia, and its early recognition can aid in the identification of preclinical and prodromal states, even before cognitive decline. Research of AD is currently focusing on detecting individuals at the earliest stages, with the aim to develop effective disease-modifying treatment approaches.Amyloid PET and beta-amyloid levels in the cerebrospinal fluid are useful biomarkers for the preclinical stages of AD [35]; however, the additional use of clinical early markers of cognitive impairment might enhance the pool of individuals atrisk, whomay be possible candidates for relative clinical studies. In this regard, defining MBI and its criteria within the context of research in neurodegenerative diseases is of paramount importance.

Although the frequency, clinical correlates, and underlying pathophysiology of various individual NPSs have been widely studied in PD, it is only recently that research began to focus specifically on MBI, as a global indicator of the emergence of sustained behavioral symptoms in older individuals. In this context, a growing body of evidence suggests that MBI is highly prevalent in non-demented PD patients, also being associated with disease severity, motor disability, and cognitive decline. Neuroimaging studies have revealed that MBI in patients with PD may be related todistinct patterns of brain atrophy, altered neuronal connectivity, and distribution of DAT depletion, shedding more light on its pathophysiological background. Genetic studies in PD patients have also shown that specific SNPs may be associated with MBI, paving the way for future research in this field. To the best of our knowledge, there is no relative review aiming to integratethe existing literature on MBI in PD.

In this narrative review, we summarize and critically discuss the emerging evidence on the frequency, associated clinical and genetic factors, as well as neuroanatomical and neurophysiological correlates of MBI in PD, aiming to elucidate the underlying pathophysiology and its potential role as an early marker of cognitive decline particularly in this population. In addition, we aim to identify research gaps, and propose novel relative areas of interest that could aid in our better understanding of the relationship of this newly defined diagnostic entity with PD. Our deeper understanding of the longitudinal trajectory of NPSs and their relationship with cognitive decline in PD is of paramount importance, contributing also to the stimulation of further research for prompt recognition and therapeutic intervention.

For our literature review, we followed a systematic approach, searching in MEDLINE and Scopus databases for articles investigating MBI in PD, in terms of clinical, genetic, neuroanatomical, and neurophysiological aspects, with no time restrictions. The search was performed between September 2023 and November 2023. We used the terms “mild behavioral impairment”, “MBI”, “neuropsychiatric symptoms”, “behavioral symptoms”, “depressive”, “depression”, “anxiety”, “affective”, “emotional”, “mood disorders”, “motivational”, “apathy”, “impulse control”, “hallucinations”, “psychotic”, “psychosis”, “delusions”, “mild cognitive impairment”, “MCI”, “cognitive decline”, “dementia”, and “Parkinson’s disease” in different combinations. The search results were screened in relation to title and abstract, and relevant articles were read in their full form. By using the snowballing process, we screened the bibliography of the relevant articles for additional studies. We primarily included studies investigating MBI in PD and its clinical, genetic, neuroanatomical, and pathophysiological correlates. However, for discussion purposes, we also included studies investigating individual NPSs in PD.

## 2. MBI Definition and Assessment

According to the NPS Professional Interest Area (PIA) of the International Society to Advance Alzheimer’s Research and Treatment (ISTAART)‒Alzheimer’s Association (AA) criteria of MBI, NPSs should be present for at least six months in individuals above the age of 50 years, not attributed to a formal psychiatric diagnosis such as major depression, schizophrenia, orgeneralized anxiety disorder, and they should constitute a clear change from the usual personality or behavior as observed by the patient, or a suitable informant [36]. MBI symptoms are subdivided into five domains, including decreased motivation, impulse dyscontrol, abnormal perception or thought content, affective dysregulation, and social inappropriateness [36]. The intensity of NPSs can vary from mild to severe, but they should be adequate enough tocause at least minimal impairment tointerpersonal relationships, workplace performance, or other social aspects [36]. However, the individual should be independent in daily life activities, needing no or minimal assistance. Although dementia excludes the co-occurrence of MBI, MBI may coexist with subjective cognitive decline (SCD) or mild cognitive impairment (MCI).

The MBI Checklist (MBI-C) has been recently developed as an instrument to identify emergent and sustained NPSs in older adults in the community, based on the criteria of MBI, particularly regarding the reference range of 6 months, the appearance of impactful NPSs in later life, and the exclusion of pre-existing comorbid psychiatric illness that is independent of the neurodegenerative process (freely available at https://mbitest.org/ (accessed on 10 November 2023)) [30]. It is a simple two-page rating scale completed by a suitable informant in theform of a questionnaire, consisting of 34 items which are organized in the five MBI domains as described above. In particular, it includes (a) six items for decreased motivation, evaluating cognitive, emotional, and behavioral apathy, (b) six items for affective dysregulation, evaluating low mood, guilt, anhedonia, hopelessness, panic, and worry, (c) twelveitems for impulse dyscontrol, evaluating aggression, agitation, recklessness, impulsivity, as well as abnormal reward and reinforcement, (d) five items for social appropriateness, assessing empathy, sensitivity, and tact, and (e) five items for abnormal perception or thought content, assessing grandiosity, suspiciousness, as well as visual and auditory hallucinations [30]. For each question, if the answer is “Yes”, a severity scale follows [1 (mild), 2 (moderate), 3 (severe)] [37]. The MBI-C can produce domain-specific and also an overall score, classifying individuals with MBI or not, by using validated cut-off points [38].

Since the construct of MBI-C is relatively new, several epidemiological studies have used other older scales for defining MBI, such as the Neuropsychiatric Inventory (NPI) and its shorter version, NPI Questionnaire (NPI-Q). The NPI measures the frequency and severity of twelve behavioral symptoms via a composite score from zero to twelve. Sheikh and colleagues have developed an algorithm that uses ten behavioral domains of the NPI, which could correspond to the five MBI symptoms as follows: (a) apathy/indifference in the NPI: decreased motivation, (b) depression/dysphoria, anxiety, and elation/euphoria in the NPI: affective dysregulation (c) agitation/aggression, irritability, aberrant motor behavior, liability in the NPI: impulse dyscontrol, (d) disinhibition in the NPI: social inappropriateness, and (e) delusions and hallucinations in theNPI: abnormal perception or thought content [39]. As sleep, appetite, and eating behavior disturbances, which constitute the neuro-vegetative domain, are not included in the MBI definition, these NPI items are not used for characterizing MBI. MBI diagnosis is made if the NPI score is at least one in at least one of the five MBI domains. For fulfilling the criterion of the minimum duration of six months, a relevant modification in the reference range of symptom duration in the NPI can also be applied [39].

A validation study in a cognitive clinic has demonstrated significant correlation between global total scoreson the NPI-Q and MBI-C, although there was a lower correlation for subjective cognitive decline (SCD), compared to MCI [40]. Even thoughthe MBI-Cis a tool aiming to capture pre-dementia states, the MBI-C has also been used in populations with dementia. In this regard, the MBI-C has shown good test‒retest reliability, internal consistency, and inter-rater reliability in validation studies investigating the MBI-C translated in Chinese [41,42]. Concerning MBI diagnosis, cut-off points of total MBI-C scores of 6.5 in MCI and 8.5 in SCD have shown good sensitivity and specificity, although alternative cut-off points have also been used [43].

Although the MBI-C is a valuable instrument for evaluating MBI, there are some possible challenges and limitations related to its use. Optimal cut-off points for populations with normal cognition, as well as for individual MBI domainshave not been determined yet [43]. Furthermore, since MBI aims to capture subtle behavioral changes, the selection of an appropriate and reliable informant plays a pivotal role in observing even mild behavioral symptoms, and different informants mightinterpret behaviors differently, resulting in inaccurate measurements. Although for MBI diagnosis, emergent and persistent NPSs should not be attributed to a prior psychiatric disease, it might be difficult to determine the psychiatric history of the patient, due to recall bias or missing information in medical records. Furthermore, because of cultural differences, the MBI-C might not effectively capture abnormal behavioral changes across different populations, since specific behaviors might be considered typical in one culture and atypical in another.

## 3. The Epidemiology of MBI in PD

**Figure 1:**
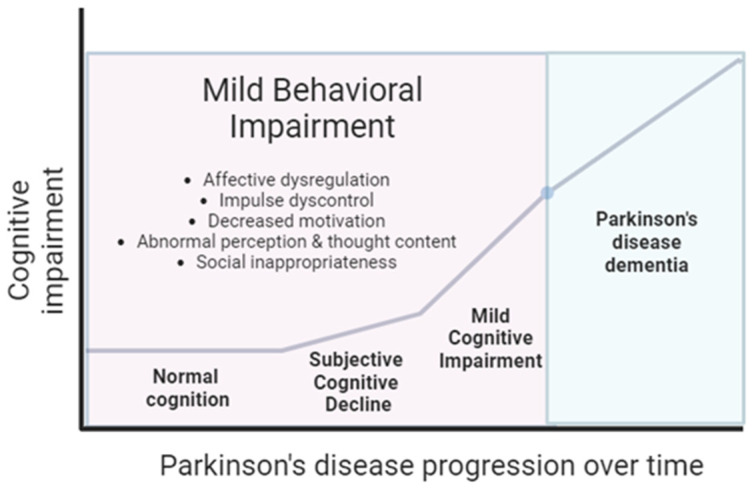
Time frame of Mild Behavioral Impairment (MBI) in Parkinson’s disease.

Several studies have estimated the prevalence of MBI in non-PD populations, in both hospital- and community-based settings (Table 1, Figure 1). A study in a memory clinic has demonstrated that the frequency of MBI, as evaluated by the NPI-Q, was about 85% and 77% in individuals with MCI and SCD, respectively [39]. Using the NPI-Q, the MBI prevalence in a community-based study was lower, approximately 43% forSCD and 49% forMCI [44]. Another prospective study defining MBI via NPI-Q evaluations at the baseline and 1year, showed that MBI prevalence was even lower, approximately 7%, 13%, and 25% in non-demented individuals without cognitive deficits, mild cognitive deficits, and moderate cognitive deficits, respectively [45]. MBI frequency in primary care health centers using the MBI-C was about 6% and 14% forSCD and MCI, respectively [37,46]. A recent meta-analysis indicated that affective dysregulation and impulse dyscontrol are the most frequently affected MBI domains, followed by decreased motivation and social inappropriateness [47].

Recent studies have shed light on the prevalence of MBI and its specific domains affected in PD. The Parkinson’s Disease Cognitive Impairment Study (PACOS) including 429 non-demented PD patients is a hospital-based, cross-sectional study in southern Italy, aiming to investigate the burden, biomarkers, and progression of MCI in PD [48]. In this study, MBI, characterized via the modified NPI-Q algorithm, appeared in about 84% of non-demented PD patients [48]. MBI appeared in about 32% of 275 drug-naïve non-demented individuals with PD in another study, in which MBI was determined when there were deficits in at least two NPS items in the NPI-Q (either two impaired items in one domain, or one impaired item in two domains) [49]. MBI frequency was approximately 33% in another study including 60 non-demented PD patients, by using the MBI-C [50]. A previous study in community-based PD clinics has shown that about 72% of PD patients with normal cognition and 79% of those with MCI reported at least one NPS as evaluated by the NPI, without the modification of 6 months [51]. At least one NPS has been also reported in about 64% of non-demented PD patients in a hospital-based study, as evaluated by the Mini-international neuropsychiatric interview (MINI) [52].

As previously demonstrated in non-PD populations, the different scales, time frames and methods used for MBI characterization may significantly affect the consistency and comparability of relevant epidemiological studies, and subsequently the estimation of theprevalence of MBI in PD. In particular, the NPI is a tool constructed specifically for patients with dementia, including questions like wandering, purposeless activities, and resistance to care [36]. Although the NPI covers various NPSs, it uses a severity scoring system that might not be sensitive enough to captureearly-stage, mild behavioral alterations associated with MBI. In contrast, the MBI-C might emphasize early, subtle changes and provide a more graded approach to assessing milder behavioral symptoms.On the other hand, the MBI-C was designed for ascertaining MBI among community-dwelling, functionally independent individuals, thereby providing better specificity [30]. The use of the NPI without the modified time frame of six months may result in an overestimation of MBI frequency, since individuals might report transient, episodic, or reactive behaviors that donot reflect a consistent impairment. Shorter time frames could also include behavioral variations within the spectrum of normal fluctuations, which may not represent a clinically significant alteration. On the other hand, extending the assessment period to include baseline and 1-year evaluations using the NPI, as well as the necessity of two NPS items within the NPI to ascertain MBI, may result in reduced sensitivity. The use ofdifferent diagnostic thresholds may additionally limit the comparability of the relevant studies, with higher thresholds possibly resulting in the underestimation of MBI prevalence.

In addition, hospital-based PD cohorts may contribute to selection bias, since atypical cases or patients with more frequent and severe NPSs might have been included in these studies, resulting in the overestimation of NPSs and MBI. Given also the fluctuating nature of cognitive and psychiatric symptoms in Lewy body diseases, including PD, the extended time frame becomes even more important for assessing MBI in these cases.

In contrast to the varied findings on MBI prevalence, it has been consistently shown that the most frequently affected MBI domains in PD are affective dysregulation, decreased motivation, and impulse dyscontrol, independently of the scale and method used for MBI characterization [48,49,50]. In line with this evidence, a hospital-based study demonstrated that—excluding sleep and appetite disturbances—depression and anxiety were the most common NPSs in PD as assessed by the NPI, affecting about 55–70% of non-demented PD patients, followed by apathy, irritability, and agitation/aggression [53]. Another study in community-based PD clinics has indicated that the most frequent NPSs were anxiety, depression, and irritability in PD patients with normal cognition, while apathy, anxiety, and depression were the most common ones in PD patients with MCI [51]. Given the limited relative evidence, it would be interesting for future studies to investigate the potential differences in the frequency of MBI and MBI domains in PD patients with normal cognition and MCI.

## 4. The Relationship of MBI with Disease Stage and Motor Impairment in PD

**Figure 2:**
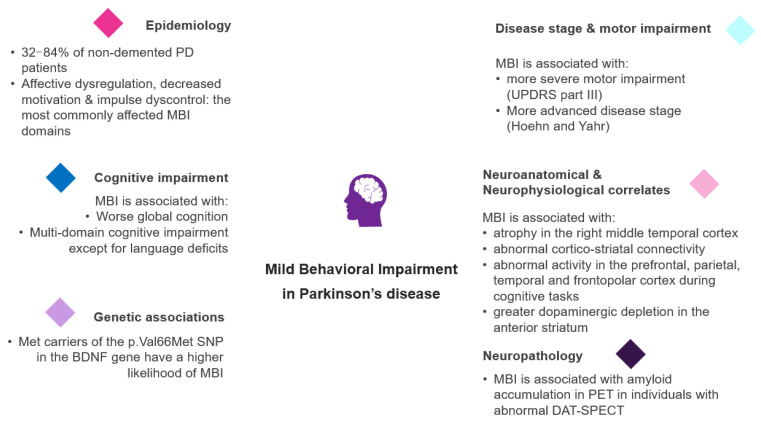
Mild behavioral impairment in Parkinson’s disease: epidemiology, associations with cognitive impairment, and genetic, neuroanatomical, neurophysiological and potential neuropathological correlates.

It has been consistently shown that MBI is associated with more severe motor impairment and higher disease stage in patients with PD (Figure 2). More specifically, PD patients with MBI display higher UPDRS part III (motor) scores compared to those without MBI [48,49,50,54], even in cases with similar disease duration between the two groups [50]. In the PACOS study, MBI was associated with higher Hoehn and Yahr stage in PD patients with a disease duration of less than a year [48]. Importantly, total MBI-C scores were also positively correlated with UPDRS part III scores in two studies [55,56]. It has been previously shown that NPSs appear more commonly as the disease progresses, and they are also related to motor impairment. In this regard, NPSs, as represented as NPI total scores, have been associated with disease duration, Hoehn and Yahr stage, UPDRS part III scores, and axial disability score in samples consisting of PD patients with normal cognition, MCI, and dementia [51,57]. Moreover, another study has demonstrated that a cluster of NPSs including irritability, agitation, disinhibition, and psychosis was associated with increased UPDRS part III scores in PD [57]. Higher motor severity at the baseline has been correlated with longitudinal worsening of NPI scores in another cohort of newly diagnosed PD patients [58]. Therefore, MBI may be associated with higher motor disability in patients with PD. Further evidence is needed in order to clarify whether MBI is related to a more rapid disease progression, and if a more severe motor disability at PD diagnosis is linked to a higher probability of MBI occurrence in PD.

Concerning the different MBI domains, impulse dyscontrol, including aberrant motor behavior, irritability, and agitation, was significantly more frequent in patients with a disease duration of more than one year, compared to the newly diagnosed patients in the PACOS study [48]. The frequency of each NPS also increased with higher Hoehn and Yahr stages for all MBI domains with the exception of affective dysregulation, although significant results were only demonstrated for abnormal perception and social inappropriateness [48]. The increased disease duration and severity are significant risk factors for the development of psychotic manifestations in PD patients [13]. Furthermore, scores in apathy scales have been correlated with UPDRS part III scores and higher Hoehn and Yahr stages in patients with PD [59]. The fact that anxiety and depression are very common non-motor manifestations of PD even in early stages [52] might explain these results. It has also been hypothesized that the increasing MBI frequency as the disease progresses might be explained by the psychological reaction to the increasing motor disability. However, the lack of an association between depressive symptoms and greater motor deficits in the abovementioned study [48] argues against this hypothesis(Figure 2).

## 5. The Link between MBI and Cognitive Impairment in PD

NPSs are a core feature of PDD, and PD patients with dementia display more frequent NPSs compared to those with MCI or normal cognition [60]. Before the development and wider use of the new construct of MBI, most studies in PD had focused on the relationship between individual NPSs with either global cognition or specific cognitive deficits, especially executive dysfunction. Executive dysfunction reflecting cortico-striatal impairment is very commonly observed in PD, even at early stages [61]. In this context, executive impairment in PD patients has been consistently related to several individual NPSs, including apathy [62], depression [63], anxiety [64], visual hallucinations [65], and impulse dyscontrol [66]. Furthermore, total NPI scores have been associated with executive deficits in non-demented PD patients [67]. In addition, anxiety in newly diagnosed PD patients has been linked to greater cognitive deficits and particularly memory impairment [68]. Non-demented PD patients with apathy also exhibit higher conversion rates to PDD compared to those without apathy during a follow-up period of 18 months [15], and apathy is also an important NPS discriminating PD patients with MCI from those without cognitive impairment [51]. Depressive symptoms as assessed by the NPI and Geriatric Depression Scale (GDS) were also related to a higher risk of MCI development among newly diagnosed PD patients in a longitudinal study [69].

Emerging evidence has demonstrated that MBI is associated with cognitive impairment in PD. In this context, MCI has been shown to be significantly more common in PD patients with MBI, compared to those without MBI in three studies [50,54,70]. On the other hand, in the PACOS study, MBI was only marginally associated with MCI in PD, and the association was lost after stratification by disease duration (cut-off of one year), as well as after controlling for age, sex, and years ofeducation [48]. The differences in the abovementioned studies could be at least partially explained by the different measurements used for characterizing MBI. The presence of just one behavioral symptom across the 12 domains of the NPI was used as a cut-off for determining MBI [48], which, as analyzed above, might have contributed to the lower specificity.

The relationship between MBI with global and domain-specific cognitive functions in PD has also been explored. In this context, it has been shown that compared to PD patients without MBI, those with MBI displayed worse global cognitive function, as assessed by Montreal Cognitive Assessment (MoCA) [50,54] and the Korean version of the Mini-Mental State Examination (MMSE) [49]. In accordance with this, the total MBI-C score has been negatively correlated with (MoCA) scores in three studies [50,55,56]. In regard to specific cognitive domains, MBI, as assessed by the MBI-C, was related to worse performance in each of the five cognitive domains (attention, executive function, language, memory, and visuospatial abilities), and the total MBI-C score was negatively correlated with all z scores, with the exception of language after adjustment for UPDRS part III [50]. Within the subgroup of PD-MCI patients, the four distinct MCI subtypes (amnestic multiple, non-amnestic multiple, amnestic single, non-amnestic single) did not significantly differ between PD-MBI and PD-non-MBI, while MBI was associated with worse attention, executive function, and memory [50]. In the PD-MCI subgroup, scores forlanguage did not significantly differ between PD-MBI and PD-non-MBI, while visuospatial abilities were worse in PD-MBI only without adjustment for UPDRS part III [50]. In agreement with this evidence, the PD-MBI group displayed reduced scores in memory, attention, and visuospatial ability, compared to the PD-non-MBI group in another study [54]. Furthermore, MBI was associated with lower scores in memory and Stroop color reading tests that assess frontal dysfunction, while scores forlanguage did not significantly differ between PD-MBI and PD-non-MBI groups [49]. Therefore, MBI in PD seems to be related to worse global cognition and a rather multiple-domain cognitive impairment, with the exception of language.

It is already known that in non-PD populations with MCI, the presence of MBI is associated with greater cognitive impairment and increased rates of conversion to dementia [31,32,33,34]. Based on the abovementioned evidence, MBI can be speculated to represent an early marker of cognitive decline in PD, possibly reflecting a higher risk of conversion to PDD among PD patients with MCI. As described above, MBI was associated with worse global cognition, executive, memory, and visuospatial deficits in PD patients with MCI [50], thereby potentially reflecting a more severely affected endophenotype that could possibly progress more rapidly to PDD. According to the hypothesis of the dual-syndrome, memory and visuospatial dysfunctions, which are related to the posterior and temporal brain regions, may predict more effectively the risk of dementia, compared to the executive fronto-striatal dysfunction in PD [71]. It has been previously indicated that depression, irritability, and apathy are more common in amnestic MCI compared to the non-amnestic MCI in non-PD populations [72], further supporting the relationship between NPSs and memory function. Collectively, this evidence suggests that in PD, MBI, as a global indicator of emergent and sustained NPSs, may be associated with worse global and multiple-domain cognitive impairment except for language, and it might also represent a non-cognitive early clinical “marker” of cognitive decline in PD patients. However, future large longitudinal studies are needed to confirm this hypothesis.

## 6. The Genetic Background of MBI in PD

Brain-derived neurotrophic factor (BDNF) plays a pivotal role in neuronal cell survival, differentiation, and synaptic plasticity, also being implicated in multiple signaling pathways. BDNF concentration has been found to be reduced in the substantia nigra of patients with PD [73]. The p.Val66Met (G758A, rs62265) single-nucleotide polymorphism (SNP) in the exon 11 of the *BDNF* gene, resulting in the substitution of a valine (Val) with a methionine (Met) residue at position 66, has been related to cognitive impairment in PD [74]. This substitution, located in the BDNF precursor protein, reduces the extracellular BDNF release, thereby limiting its availability in the central nervous system. In addition, the Met allele has been associated with a higher risk of depression in geriatric non-PD populations, and there is some evidence that it may also be linked to schizophrenia and post-traumatic stress disorder [75]. Lower serum BDNF levels have also been related to depression [76]. Given its relationship with both PDD and psychiatric conditions, it has been hypothesized that the p.Val66Met polymorphism may be related to MBI in PD.

In this regard, a recent study among 146 PD patients has shown that MBI, as evaluated by the MBI-C, is associated with carrying at least one Met allele of the p.Val66Met SNP in the *BDNF* gene [55]. In particular, Met carriers had higher total MBI-C scores and a two times higher likelihood of having MBI, compared to Val homozygotes, after controlling for UPDRS part III and MoCA scores [55]. PD patients carrying the Met allele also exhibited more severe affective dysregulation and aberrant perception, compared to Val homozygotes [55]. On the other hand, a previous study by Cagni and colleagues in 104 PD patients demonstrated that depression and anxiety, assessed by the Beck Depression Inventory (BDI) and the Beck Anxiety Inventory (BAI), respectively, were associated with the Val allele [77]. These different results might be explained by the different population composition and the different methods of NPS evaluation. In the study of Cagni and colleagues, a relatively large proportion of the sample had a family history of PD and early-onset disease [55,77], potentially leading to selection bias. Depression has been shown to be more common in patients with early-onset PD [78], as well as in LRRK2 G2019S carriers [79]. Given the additional clinical differences between early-onset and late-onset PD, such as in regard to levodopa-induced motor complications [80], further studies should also investigate the relationship between MBI and PD-related cognitive impairment separately in these PD subgroups. Furthermore, in the study by Cagni and colleagues, NPSs were assessed usingBDI and BAI, two self-reporting tools referring to the last two and four weeks, respectively, compared to the MBI-C that refers to the last 6 months [55,77]. The different time frames and tools might have also contributed to the different results.

## 7. Neuroanatomical Correlates of MBI in PD

Given that MBI is associated with a higher risk of cognitive impairment, it has been hypothesized that MBI may be related to greater brain atrophy in patients with PD. In this context, PD patients with MBI exhibited thinning and reduced volume in the right middle temporal cortex, compared to those without MBI, and the total MBI-C score was correlated with the reduced cortical volume in this region [50]. Furthermore, compared to healthy controls, the PD-MBI subgroup showed cortical changesin the left parahippocampalarea, right precuneus, lateral frontal pole, and right lingual gyrus [50]. Concerning subcortical regions, no significant associations were detected [50]. It has been previously demonstrated that NPSs are related tocortical atrophy in the temporal and frontal areas in PD. In particular, a more rapid cortical thinning in the temporal region has been longitudinally related to depressive symptoms in PD patients [81]. Agitation, aggression, and total NPI score have been associated with decreased temporal cortical thickness in patients with PD [82]. PD patients with visual hallucinations displayed cerebral blood flow alterations in the temporal region [83]. Atrophy in the frontal areas is also frequently demonstrated in PD patients with apathy, visual hallucinations, depression, and impulse dyscontrol [84]. PD patients with NPSs, as assessed by the NPI [82] and the Cambridge Behavioural Inventory–Revised [85], have been shown to have greater prefrontal atrophy. These results suggest the pivotal role of both the temporal and frontal cortical areas in a wide range of NPSs in PD.

Importantly, PD patients with MCI or dementia display more pronounced cortical atrophy in the temporal region compared to those with normal cognition [86,87]. Brain atrophy in this area is also associated with deficits in memory and visuospatial abilities in PD [88,89], and MCI in PD has been related to more rapid cortical thinning in the lateral temporal area in a longitudinal study [90]. Lower thickness in the temporal cortex at the baseline has also been linked to progression to MCI in PD patients with normal cognition within 18 months [91], and PD patients who longitudinally developed dementia also displayed reduced cortical temporal thickness compared to those who did not [92]. This evidence combined with the association between MBI and temporal cortical atrophy in PD further highlight the promising role of MBI as a potential indicator of cognitive decline in PD.

## 8. The Relationship between MBI and Functional Connectivity in PD

Cortico-striatal connectivity plays a major role in both cognitive and behavioral symptoms in PD, also being hypothesized to represent a shared dysfunctional network between these two conditions. Individual NPSs have been consistently associated with impaired striatal function and the cortico-striatal network in PD, including depression, anxiety, and apathy [93]. The underlying impaired neurotransmitter systems have been proposed to involve both dopaminergic and non-dopaminergic pathways, including noradrenergic and serotonergic systems [94]. The connectivity between the striatum and default mode network is disrupted in PD patients [95], as well as in depression and where there is a high risk of psychosis in non-PD populations [96]. Cognitive flexibility has also been associated with enhanced striatum-default-mode network connectivity in healthy individuals [97]. Lower striatal-saliency network connectivity has been previously related to decreased disease severity in PD, as evaluated by the total UPDRS score [98], which incorporates the degree of motor, functional, cognitive, and behavioral deficits.

In this context, it has been hypothesized that MBI in PD may be linked to abnormal cortico-striatal functional connectivity. Indeed, PD patients with MBI, as assessed by MBI-C, displayed reduced connectivity between the caudate nucleus and both the default mode and saliency networks, compared to those without MBI and healthy controls, after controlling for MoCA and UPDRS part III [54]. Increased total MBI-C scores were particularly associated with reduced connectivity between the head of the left caudate nucleus andthe left middle frontal gyrus and dorsal anterior cingulate region [54]. Higher total scores of the MBI-C were also correlated with lower connectivity between the head of the right caudate nucleus and the precuneus, anterior cingulate region, and left supramarginal cortex, as well as higher connectivity with the right cerebellar hemisphere and left hippocampus [54]. Finally, the connectivity between the caudate nucleus and precuneus was independently related to global cognitive and behavioral performance [54]. These connectivity patterns have been observed in impaired limbic and associative striatal loops, which are related toboth emotional and cognitive function [99]. Collectively, it seems that MBI in PD is associated with impaired cortico-striatal connectivity, while the dysfunctional precuneus-caudate network might underlie both global cognitive and behavioral impairment, suggesting that it could be specifically associated with the relationship between MBI and cognitive decline in PD.

PD can be considered as a set of three types of clinical symptoms, including motor, cognitive, and neuropsychiatric domains. These categories exhibit varying degrees of correlation in terms of their frequency, severity, and associated factors. For instance, more severe cognitive impairment is related to worse NPSs and motor function [53]. Based on this concept, a recent study aimed to identify the common and unique influences of cognitive, neuropsychiatric, and motor symptoms on neural connectivity patterns via commonality analysis [56]. In thisstudy, the caudate nucleus was demonstrated to be the “hub” of the functional connectome underlying these three clinical domains [56], which agrees with previous evidence indicating that the caudate nucleus is implicated in motor, cognitive, and emotional processes in PD [100]. NPSs, as evaluated by the MBI-C, accounted for the neural connectivity in two circuits, the one involving the caudate nucleus, right dorsolateral prefrontal cortex, and right inferior parietal area, and the other involving the caudate nucleus and dorsal anterior cingulate area [56]. This evidence implies that the variability in the functional connectivity in these two networks may be accounted for mainly by the unique influence of NPSs and particularly the global behavioral impairment, expressed by the MBI-C [56]. The dorsal anterior cingulate is implicated in several cognitive and behavioral functions, such as cognitive control, expression of fear, behavioral adaptation, and reward-based decision-making [101,102], while the striatal-dorsal anterior cingulate pathway plays a critical pathophysiological role in apathy in PD [103]. In addition, the right dorsolateral prefrontal cortex plays an important role in both NPSs, including depression, psychotic symptoms, and anxiety, and cognitive functions, including working memory and attention [56,104]. The right inferior parietal lobe may be implicated in multidimensional cognitive processes, including the maintenance of attentive control of tasks and the response to salient new environmental evidence [105]. Both NPSs and cognitive deficits were uniquely linked to the circuit between the caudate nucleus and medial prefrontal cortex, while the circuit between the caudate nucleus and precuneus area reflected both unique and shared effects of NPSs and cognitive deficits [56]. Hence, the variability in the caudate nucleus-precuneus connectivity seems to be represented by an overlapping effect of the MBI-C and MoCA. On the other hand, the posterior cortical connectivity was demonstrated to reflect a more complex interplay between cognitive, motor, and neuropsychiatric deficits [56]. Based on the above evidence, it has been speculated that the global neuropsychiatric impairment as evaluated by the MBI-C might be a sensitive early marker of dysfunctional fronto-striatal circuits in PD.

## 9. MBI and Associated Neuronal Activity Patterns in PD

The Wisconsin card sorting task (WCST) is a commonly utilized tool for testing executive function in PD, particularly cognitive flexibility with a set-shifting task [106]. Cognitive flexibility is considered to be represented by the number of perseverative errors during the WCST. In healthy controls, WCST performance is accompanied by activation ofthe fronto-striatal loop, with the prefrontal cortex and the caudate demonstrating activation during the planning of a set-shift, with the premotor cortex and putamen being activated during the execution of a set-shift [107]. In PD, impaired set-shifting has been previously linked to the dysregulation of fronto-striatal loops [107]. A recent study aimed to investigate the relationship between MBI and brain activity patterns in PD patients and healthy controls, via functional Magnetic Resonance Imaging (fMRI) during a modified computerized version of the WCST [70]. Healthy controls and PD patients without MBI demonstrated enhanced activity in the ventrolateral and dorsolateral prefrontal cortex, posterior parietal region, thalamus, and caudate nucleus, while PD patients with MBI displayed no significant activation in these regions [70]. Furthermore, PD patients with MBI displayed reduced activation levels in the right dorsolateral prefrontal cortex and inferior parietal lobule while planning the set-shift, compared to PD patients without MBI and healthy controls [70]. MBI in PD was associated with higher rates of perseverative errors compared to healthy controls, while the rate of perseverative errors was correlated with the activity in the inferior parietal lobule and dorsolateral frontal cortex during the set-shift planning [70]. The dorsolateral and ventrolateral prefrontal cortex, as well as the posterior parietal region are majorly implicated in the adaptive behavioral control during several cognitive tasks. MCI has been associated with reduced neuronal activity in the posterior parietal and prefrontal cortex in early PD [108]. In addition, the disruption of the frontoparietal control network has been implicated in various NPSs in non-PD populations, including late-life depression and social anxiety disorders [109]. Collectively, this evidence supports the hypothesis that MBI is related to cognitive flexibility impairment in PD, possibly caused by the dysregulation of the fronto-parietal control network [70].

Given the association of MBI with cognitive impairment, the abovementioned study investigated the relationship between MBI and altered neuronal activity after controlling for cognitive function using MoCA scores [70]. The lower neuronal activity in the dorsolateral prefrontal cortex in the presence of MBI was lost after adjustment for cognitive function, while the lower activity in the inferior parietal region remained significant [70]. These differences suggest that the activation of the inferior parietal cortex depends to a greater extent on behavioral impairment compared to the dorsolateral prefrontal cortex, whose altered activation patterns may also be associated with cognitive decline [70]. In agreement with this hypothesis, the prefrontal cortex is implicated in the evaluation of goal-directed attention, while the posterior parietal area is involved in the capacity of working memory and the sustained attention during the preparation of upcoming stimuli [110], suggesting that these brain areas playdiverse roles during the executive function [70]. Moreover, total MBI-C scores have been significantly correlated with the activation levels in the postcentral region and left supramarginal gyrus during the execution of set-shift, but only after controlling for MoCA. Given these results, it has been suggested that the higher activity in the parietal cortex in the presence of behavioral impairment in PD might reflect a compensatory mechanism that exists only if cognition is normal [70]. However, future research is needed to elucidate the different neuronal networks and mechanisms underlying the cognitive and behavioral deficits in PD.

In addition to the involvement of the prefrontal and inferior parietal cortex in MBI in PD, altered neuronal activity has also been observed in the medial temporal area. In particular, during the planning of the set-shift in the study described above, the PD-MBI group didnot show the deactivation in the hippocampus that was illustrated in the PD-non-MBI group and healthy controls [70]. In accordance, the total MBI-C score was correlated with the decreased hippocampal deactivation [70]. Interestingly, disrupted fronto-striatal neuronal connectivity and decreased hippocampal deactivation have been correlated with lower levels of dopamine in healthy controls while performing the WCST [111]. Lower hippocampal activity has been previously demonstrated while shifting from the old rule to the generation of new rules in comparison to the maintenance of the old rule during the performance of a set-shifting task [112]. Furthermore, during a set-shifting task, an indirect interaction has been revealed between the caudate nucleus and the hippocampus [112]. It is considered that the hippocampal deactivation while performing cognitive procedures that require attention may prevent the interference of ruminations and stimulus-independent memories, including previous trials in the set-shift task with current trials [70]. Therefore, MBI might be associated with both hippocampal and frontal executive dysfunctions in PD.

In the same study, during the execution of set-shift, lower activation of the left frontopolar region was observed in PD patients with MBI compared to healthy controls [70]. The executive process of constant adding and deleting appropriate content in the working memory has been related to the left frontopolar cortex [113]. The more perseverative and non-perseverative errors together with the decreased activation of the frontopolar region in PD patients with MBI compared to healthy controls may suggest executive dysfunction, especially the updating and inhibition processes [70]. Given also the decreased activation of the dorsolateral prefrontal cortex while planning the set-shift, this evidence suggests that MBI in PD may be linked to multiple disrupted prefrontal cognitive tasks underlying set-shifting.

## 10. MBI and Nigrostriatal Dopaminergic Degeneration in PD

MBI has been associated with reduced dopamine transporter (DAT) availability in the anterior caudate nucleus and anterior putamen, as evaluated by the N-(3-[18F]fluoropropyl)-2β-carbomethoxy-3β-(4-iodophenyl) nortropane (18F-FP-CIT) PET, after adjustment for age, sex, and years of education [49]. The head of the caudate nucleus is involved in emotional and cognitive processing [114], and the anterior putamen also plays an important role in behavior [115]. Apathy and depressive symptoms have been associated with higher dopaminergic depletion in the caudate nucleus of PD patients [18,116]. Reduced DAT availability in the left anterior putamen has also been correlated with more severe anxiety and depressive symptoms [117]. Notably, DAT availability in the caudate nucleus, anterior putamen, and ventral striatum has been associated with attention, working memory, executive, and visuospatial functions in a cohort of non-demented PD patients, while no associations were detected for the DAT availability in the posterior putamen [118]. Hence, early neurobehavioral impairment in PD may be linked togreater dopaminergic deficit in the anterior striatal region. Future studies adjusting for cognitive function may shed more light on the relationship between MBI and striatal subregions in PD.

In the same study, MBI was associated with higher scores in UPDRS part III in drug-naïve PD patients, after controlling for DAT availability in the posterior putamen [49]. These results suggest that MBI may be linked with worse motor impairment at similar levels of nigrostriatal degeneration, possibly representing a distinct disease endophenotype. In addition to dopaminergic dysfunction, dysregulation of non-dopaminergic neurotransmitter systems, including glutamate, acetylcholine, and noradrenaline, have been proposed to underlie the axial motor deficits in PD [119]. Motor impairment in PD manifests after the death of about 50% of the dopaminergic neuronal cells in the substantia nigra, and serotoninergic innervation is observed in the pre-motor stages of PD [120], possibly acting as a compensatory mechanism for the dopaminergic loss. Given these findings, it has been hypothesized that MBI might reflect a limited capacity of the non-dopaminergic systems to compensate for the dopaminergic neuronal loss in the substantia nigra in PD.

## 11. Neuropathological Correlates of MBI in PD

A recent study has indicated that among non-demented individuals with abnormal DAT-SPECT, scores in the MBI domain of abnormal perception and thought content were higher in individuals with positive amyloid positron emission tomography (PET), compared to those with negative amyloid PET, while total MBI scores did not significantly differ between these groups [121]. In accordance with this, reduced cerebrospinal fluid (CSF) beta-amyloid levels at the baseline have been associated with a higher risk of psychosis in PD [122], and early-onset formed hallucinations have been linked to cortical thinning in parietal, occipital, and frontal cortex, as well as decreased hippocampal volume. These findings suggest that psychotic symptoms in PD may be associated with amyloid pathology. However, the relationship between MBI and Lewy body or Alzheimer-type neuropathology in PD is unknown, and future longitudinal and neuropathological studies may aid in clarifying whether MBI increases the risk for a specific dementia subtype in PD.

**Table 1:** Studies investigating the frequency, associated clinical factors, and genetic, neuroanatomical, and neurophysiological correlates of mild behavioral impairment in Parkinson’s disease.

Study	Study Type	Country or Region	Study Participants	PD & MCI Diagnosis	MBI Characterization	Main Results
[48]	Cross-sectional, hospital-based, data derived from PArkinson’s Disease COgnitive Impairment Study (PACOS)	Italy	Patients with PD without dementia, with or without MCI (*n* = 429)	PD: United Kingdom PD Society Brain Bank criteria MCI: MDS Task Force, Level II criteria	NPS assessed by NPI (algorithm proposed by Sheikh and colleagues, modified reference range for 6 months) Functionality assessed by BADL (no or minimal impairment was included)	MBI appeared in 84.1% of non-demented PD patients Affective dysregulation, decreased motivation, and impulse dyscontrolwere the most common MBI symptoms Impulse dyscontrol was significantly more frequent in patients with a disease duration of more than one year MBI was not significantly associated with MCI in both subgroups (disease duration of more or less than one year) LED was not associated with MBI in both PD subgroups (disease duration of more and less than one year) after multivariate analysis
[50]	Cross-sectional	Canada	Patients with PD without dementia, with or without MCI (*n* = 60), and age- and sex-matched healthy controls without MCI (*n* = 29)	PD: United Kingdom PD Society Brain Bank criteria MCI: MDS Task Force, Level II criteria	MBI-C (cut-off point: 7.5)	MBI appeared in 33.3% of non-demented PD patients and 0% in healthy controls MBI was associated with worse global cognitive function in PD (MoCA, global score), and lower performance in each of the five cognitive domains (z scores for attention, executive function, language, memory, and visuospatial abilities) Global and domain-specific cognition did not differ between healthy controls and PD patients without MBI The total MBI-C score was negatively correlated with global cognition and z scores in all cognitive domains, with the exception of language after adjustment for UPDRS part III MCI was more frequent in PD patients with MBI compared to those without MBI (75% versus 42.5%, respectively) MBI was associated with thinning and reduced volume in the right middle temporal cortex in PD The total MBI-C score was negatively correlated with the reduced volume in the right temporal cortex PD-MBI subgroup displayed thinning in the left parahippocampal cortex, reduced surface and volume in the right precuneus cortex, andreduced volume in the lateral frontal pole and right lingual cortex, compared to healthy controls
[49]	Cross-sectional	South Korea	Drug-naïve newly diagnosed PD patients without dementia (*n* = 275)	PD: United Kingdom PD Society Brain Bank criteria	NPSs assessed by the NPI with the modified reference range of 6 months MBI diagnosis was determined when there were deficits in at least two NPS items (either 2 impaired items in 1 domain, or 1 impaired item in 2 domains)	MBI existed in 32.4% of PD patients atthe time of diagnosis Affective dysregulation, decreased motivation and impulse dyscontrolwere the most frequently affected MBI domains MBI was associated with higher scores in UPDRS part III MBI was associated with lower scores in memory and Stroop color reading tests that assess frontal dysfunction MBI was associated with reduced DAT availability in the anterior caudate and anterior putamen
[55]	Cross-sectional	Canada	PD patients (*n* = 146)	PD: United Kingdom PD Society Brain Bank criteria	MBI-C (cut-off point: 8)	PD patients with the Met allele of the p.Val66Met SNP in the BDNF gene had a 2 times higher likelihood of having MBI, compared to Val homozygotes
[56]	Cross-sectional	Canada	PD patients without dementia (*n* = 74)	PD: United Kingdom PD Society Brain Bank criteria MCI: MDS Task Force, Level II criteria	MBI-C (cut-off point: 7.5)	Caudate-dorsal anterior cingulate cortex, caudate-right dorsolateral prefrontal cortex, and rightdorsolateral prefrontal cortex-right inferior parietal connectivity is driven mainly by NPSs (and not cognitive or motor symptoms) Both NPSs and cognitive deficits were uniquely linked to the circuit between the caudate nucleus and medial prefrontal cortex, while the circuit between the caudate nucleus and precuneus area reflected both unique and shared effects of NPSs and cognitive deficits The posterior cortical connectivity represents acomplex interplay of NPSs, cognitive, and motor impairment.
[54]	Cross-sectional	Canada	PD patients without dementia (*n* = 74), healthy controls without MCI (*n* = 28)	PD: United Kingdom PD Society Brain Bank criteria MCI: MDS Task Force, Level II criteria	MBI-C (cut-off point: 7.5)	MBI was associated with lower connectivity between the striatum and both the default mode and saliency networks, compared with non-MBI and healthy controls Higher MBI-C scores were associated with lower connectivity between the head of the left caudate andthe left middle frontal gyrus and dorsal anterior cingulate region Higher MBI-C scores were correlated with lower connectivity between the head of the right caudate andthe precuneus, anterior cingulate region, and left supramarginal cortex Higher MBI-C cores were associated with higher connectivity with the right cerebellar hemisphere and left hippocampus The connectivity between the caudate and precuneus was independently related to global cognitive and behavioural scores
[70]	Cross-sectional	Canada	PD patients without dementia (*n* = 59), healthy controls without MCI (*n* = 26)	PD: United Kingdom PD Society Brain Bank criteria MCI: MDS Task Force, Level II criteria	MBI-C (cut-off point: 7.5)	MBI existed in 35.6% of non-demented PD patients MCI was more common in the PD-MBI group, compared to the PD-non-MBI group (52.4% versus 26.3%, respectively). MBI in PD was associated with lower levels of activation in the prefrontal and posterior parietal cortex, and decreased deactivation in the medial temporal region as assessed by fMRI during the conducting of a modified version of the WCST, compared to PD without MBI and healthy controls
[121]	Cross-sectional	Japan	Individuals>=50 years of age without dementia (*n* = 103), divided into 5 groups (Group 1: amyloid-positive and abnormal DAT-SPECT, Group 2: amyloid-negative and abnormal DAT-SPECT, Group 3: amyloid-positive and normal DAT-SPECT, Group 4: mild cognitive impairment unlikely due to AD with normal DAT-SPECT, Group 5:cognitively normal with amyloid-negative and normal DAT-SPECT)	Prodromal PDD/ DLB defined by abnormal DAT-SPECT Preclinical/prodromal AD defined by positive amyloid PET	NPS assessed by NPI	The MBI abnormal perception or thought content scores were higher in Group 1than Group 5 The MBI total score and MBI impulse dyscontrol score in Group 4 were higher than those in Group 5.

NPS: neuropsychiatric symptoms; BADL: Basic Activities of Daily Living; PD: Parkinson’s disease; MBI: mild behavioral impairment; MCI: mild cognitive impairment; MBI-C: MBI-Checklist; UPDRS: Unified Parkinson’s Disease Rating Scale; MoCA: Montreal Cognitive Assessment; DAT: dopamine transporter; LED: levodopa equivalent dose; SNP: single-nucleotide polymorphism; fMRI: functional Magnetic Resonance Imaging; WCST: Wisconsin card sorting task; NPI: Neuropsychiatric Inventory; DAT-SPECT: dopamine transporter single-photon emission-computed tomography; PET: positron emission tomography; AD: Alzheimer’s disease.

## 12. Future Perspectives

In terms of epidemiology, the frequency of MBI in PD patients with normal cognition, subjective cognitive decline, and MCI remains to be elucidated in community-based settings. Furthermore, large longitudinal studies investigating the association between MBI and incident cognitive decline in PD are missing.

Although the abovementioned studies shed more light on the underlying pathophysiology of MBI in PD, it is still unclear which of these observed effects are pathological or compensatory in nature, and if MBI is a “primary” or “secondary” condition of PD-related neurodegeneration. Some researchers have suggested that NPSs in PD might represent a reactive situation to the motor and potentially cognitive deficits [123]. However, the fact that NPSs such as depression may appear even at prodromal stages of PD argues against this hypothesis [123]. Future work is needed, with prospective clinical and experimental studies to elucidate the pathogenesis of MBI in PD, as well as its biological relationship with cognitive impairment.

Apart from the BDNF gene, other gene mutations may also be related to MBI in PD. Catechol-O-methyltransferase (COMT) gene variants have been associated with both cognitive and psychiatric symptoms in PD [124]. The COMT Val158Met polymorphism has been linked to a faster decline in memory, visuospatial skills, and executive function [125], and may also modify the risk of apathy in PD [126]. Met/Met carriers in COMT polymorphism also display higher DAT uptake in the caudate nucleus compared to non-carriers [127]. The APOE ε4 allele has also been linked with a higher risk of visual hallucinations [128] and cognitive decline in PD [129]. Cognitive impairment and neuropsychiatric symptoms have also been associated with variations in the SNCA gene [130]. Therefore, it can be hypothesized that these SNPs may also be related to MBI in PD, and possibly modify the risk of cognitive impairment among PD patients with MBI.

In the same vein, in non-PD populations, polygenic risk scores for AD dementia have been associated with worse cognitive function in healthy individuals with MBI, but not in those without MBI [131]. Future studies should also focus on the relationship between MBI, polygenic risk scores for dementia, and cognitive decline in PD, in order to elucidate the genetic modifiers of cognitive impairment among PD patients with MBI. In addition, it would be important to clarify whetherMBI might increase the risk of specific dementia subtypes, including PDD and Lewy body dementia.

Given the abovementioned association between MBI-psychosis and amyloid accumulation in patients with abnormal DAT-SPECT, it could be hypothesized that MBI-psychosis might be associated with amnestic MCI, and subsequently with a higher risk of concurrent AD dementia in PD. The relationship between different MBI domains and MCI subtypes in PD might also be another interesting field of future research.

The identification of biomarkers for motor and non-motor progression in PD is also receiving growing attention. In this regard, higher plasma neurofilament light chain (NfL) levels have been linked to faster motor progression in PD [132]. CSF NfL levels and their longitudinal increase rate are also higher in PD patients with MCI compared to those with normal cognition [133]. In non-demented non-PD populations, MBI has been associated with increased plasma neurofilament light chains, a biomarker of axonal damage over a 2-year period, even after controlling for MCI [134]. Hence, the longitudinal relationship between MBI and plasma or CSF NfL in PD could aid in our better understanding of the impact of MBI on PD progression.

Reduced glutathione levels have been observed at the early stages of PD. Interestingly, a recent longitudinal study has demonstrated that an increase in glutathione levels was associated with less increase in the probability of prodromal PD [135]. Glutathione-related signaling pathways have been related to the development and progression of several psychiatric disorders, including depression, anxiety, and schizophrenia [136]. Further studies should also focus on the relationship between glutathione levels and MBI in PD.

Frailty has also been related to prodromal PD in community-dwelling older individuals in the Greek population [137], and several studies have shown that frailty is associated with depression among older individuals [138]. In this context, it would be interesting to investigate whetherfrailty is associated with MBI in prodromal PD.

Importantly, MBI has been associated with an increased volume of white matter hyperintensities in non-PD populations with MCI [139], and the severity of white matter hyperintensities has been associated with executive dysfunction in PD, including deficits in working memory, attention, and processing speed [140]. In this regard, no significant differences were detected between cardiovascular risk factors and MBI in patients with PD in the study investigating the relationship with MBI and DAT uptake, although patients with multiple basal ganglia lacunes or severe white matter hyperintensities were excluded [49]. Cerebrovascular disorders was also one of the exclusion criteria in the studies of Yoon and colleagues [50,70], and Ramezani and colleagues [55]. Potential relationships between MBI and white matter burden in patients with PD should also be investigated.

In non-PD populations, it has been recently demonstrated that prior head injury may be associated with the MBI-domains of affective dysregulation and impulse dyscontrol [141]. Cognitive complaints and more severe depressive symptoms have also been correlated with a higher number of prior head injuries in patients with PD [142].Neuroinflammation, abnormal protein accumulation including beta-amyloid, tau and alpha-synuclein, as well as metabolic dysregulation have been proposed as potential underlying mechanisms linking traumatic brain injury and PD [143], suggesting that these mechanisms might also play a role in PD-related MBI.

Interestingly, a recent study among older non-PD individuals without dementia indicated that MBI was associated with lower gait speed as evaluated bymeasurements of dual-task gait cost (DTGC) [144]. Gray matter volume loss has also been related to worse dual-task gait performance in comparison to single-task gait across the spectrum of Lewy body disorders [145]. Exploring the relationship between MBI and dual-task gait performance in PD may provide additional insights into the pathophysiology of PD-related MBI.

Another physiological aspect that should also be explored as a potential underlying link between MBI and cognitive impairment in PD is timing ability, involving the estimation of duration and the prediction of its consequences [146]. Timing is an important cognitive function, playing a pivotal role ininter-temporal decision-making, sensory integration, and motor coordination [146]. Abnormal timing processing has been demonstrated in several psychiatric and neurological disorders related to fronto-striatal dysfunction, including obsessive-compulsive disorder and PD [147]. In this regard, the fast-spiking interneuronshave been shown to represent the timing preference modulation in the striatum, shedding more light on the time-related pathophysiological mechanisms in PD [147], which should be further investigated in PD-related MBI.

Premorbid personality traits play a significant role in shaping an individual’s baseline behavior, which may affect the way that NPSs may manifest during a neurodegenerative process, as well as how the patient may respond, interpret, cope with, and report them. Such confounders are not usually measured in studies investigating NPSs in PD. It would be interesting for future research efforts to control for or stratify by premorbid traits, as this approach might help differentiate the impact of PD-related NPSs from that of a premorbid personality.

Till now, studies have focused on investigating MBI in manifested PD. It is already known that MBI increases the risk of cognitive decline among healthy older individuals, but it is still unknown whether MBI also increases the risk of PD. One important feature of PD is its prodromal phase, which lasts several years before the clinical onset of the disease. In a population-based study among non-demented older individuals without PD, an increased probability of prodromal PD was associated with lower cognitive function [148]. Depression is a well-characterized non-motor feature of prodromal PD, along with hyposmia, autonomic disturbances, and REM-sleep behavior disorder [149]. It has also been shown that late-life psychotic symptoms are associated with a higher risk of prodromal PD [150]. Apathy may also manifest before motor symptoms, and PD patients report decreased initiative and reduced traveling up to 8 years before PD diagnosis [151]. In addition, cognitive alterations may occur up to 6 years before the diagnosis of PD, and global cognitive impairment has now been included as an independent marker of prodromal PD, according to the recent criteria of the Movement Disorder Society (MDS) for prodromal PD [149]. However, the relationship between MBI and prodromal PD, as well as its relationship with cognitive impairment during this prodromal phase of PD remain to be explored. In this way, the incorporation of MBI into the criteria forprodromal PD may contribute to a more accurate identification of this entity, and MBI might serve as an early “marker”, offering a window for potential disease-modifying treatments prior to the manifestation of motor symptoms.

Early detection of MBI in PD may also help address NPSs early on, which may improve patients’ quality of life and overall well-being. It can also be speculated that tailored behavioral interventions could also possibly delay cognitive decline. In this context, cognitive-behavioral treatment for depression in PD has been shown to modestly improve executive function and verbal memory over a 10-week period [152]. However, the effects of interventions toward MBI in PD are unknown, and future research is needed to confirm this hypothesis. In addition, pharmaceutical modifications might be needed in PD patients with MBI for optimal management; in this regard, pramipexole might be beneficial for depressive symptoms in patients with PD with or without classic antidepressants [153,154], andpiribedilhas shown promising results for PD-related apathy [153]. On the other hand, dopaminergic agonists should be used with caution in PD patients with cognitive impairment and personality traits including novelty seeking and impulsivity, since they may induce or aggravate psychiatric manifestations, especially in these cases [153].

The potential interaction between MBI and lifestyle factors during the prodromal stages of PD should also be explored. In this regard, a Mediterranean diet has been related to a lower likelihood of prodromal PD and risk of PD and DLB in a longitudinal epidemiological study ofthe Greek population [155], and a more pro-inflammatory diet has also been linked with a higher probability of prodromal PD [156]. As adherence to a Mediterranean diet and anti-inflammatory eating patterns have also been inversely associated with depression and anxiety in non-PD populations [157,158], possible links between MBI, a Mediterranean diet, or anti-inflammatory diet patterns with PD development could also be explored.

The MBI-C can be both self- and informant-filled. However, anosognosia for non-motor symptoms has been found to be 16% in PD patients with multi-domain MCI, and greater anosognosia for cognitive impairment was also correlated with reduced depressive symptoms [159]. Since the MBI-C aims to detect subtle behavioral changes, both resources (the individual and the informant) would be rather preferred, if possible.

Furthermore, most of the abovementioned studies wereconducted on PD patients treated with dopaminergic therapy. Since dopaminergic treatment may be associated with NPSs, future studies should also focus on untreated, newly diagnosed patients with PD. In addition, adjustments should also be made for antidepressant, anxiolytic, and antipsychotic medications.

It is important to mention that research on MBI characterization, assessment methods, and underlying pathophysiology is still ongoing. Although there are some studies shedding more light on the potential mechanisms, sample sizes are often small, and longitudinal evidence is of paramount importance in investigating the relationship between MBI symptoms and neuroimaging, neuropathological, and genetic data [43]. The MBI-C needs to be validated in broader and different populations, and in diverse cultural settings. Furthermore, the different tools and diagnostic thresholds for defining MBI constitute additional challenges. Concerning the individual MBI symptoms, it has been suggested that research could firstly focus on the most common MBI domains, including affective dysregulation and impulse dyscontrol [43]. MBI detection may aid in the early identification of possible cognitive impairment, and facilitate clinical research for the development of early therapeutic interventions. It has been considered that MBI assessment may be a valuable tool especially in cases with co-occurringMBI and MCI, since these patients might have a higher risk of dementia compared to MCI or MBI alone [43].

## 13. Conclusions

Collectively, emerging evidence shows that MBI is very common in PD, affecting about 32–84% of non-demented PD patients. The differences in the relative studies highlight the importance of methodological discrepancies in terms of study settings, time frame for determining MBI, and instruments used for MBI characterization. It has been consistently indicated that the most commonly affected MBI domains in PD are affective dysregulation, decreased motivation, and impulse dyscontrol. MBI is also related to more severe motor impairment, disease stage, and a rather multi-domain cognitive impairment in PD except for language deficits, while further evidence is needed to clarify its potential as an early marker of incident cognitive decline in this population. In PD, Met carriers of the p.Val66Met SNP in the BDNF gene exhibit an increased probability of having MBI compared to Val carriers. Concerning neuroanatomical and neurophysiological correlates, MBI in PD has been associated with enhanced atrophy in the right middle temporal cortex, abnormal cortico-striatal functional connectivity, as well as abnormal activity in the prefrontal, parietal, temporal, and frontopolar cortex during cognitive tasks. Finally, MBI in PD may be linked to greater dopaminergic depletion in the anterior striatal region and amyloid accumulation.

Of note, as the concept of MBI is relatively new and is still being researched and refined, ongoing research will aid in the characterization, evaluation, as well as clinical, genetic, neuroanatomical, and neurophysiological correlates in neurodegenerative diseases including PD.

In conclusion, MBI presents an emerging and crucial area of investigation in understanding the prodromal phase of the disease, as well as the underlying neuroanatomical, neurophysiological, and genetic architecture of cognitive and neuropsychiatric impairment in PD. Future studies will aid in elucidating the potential role of MBI as an early predictive marker of cognitive decline in PD, which may also offer opportunities for risk stratification, personalized care, and targeted therapeutic interventions.

## Data Availability

Not applicable.
